# International Accreditation and Quality Medical Education

**Published:** 2013-05-30

**Authors:** Pablo A. Pulido

The Pan American Federation of Associations of Faculties (Schools) of Medicine - FEPAFEM/PAFAMS - is a non-governmental, non-profit academic organization that joins the National Associations of Medical Schools for the Hemisphere. For some countries the growth in the number of schools and colleges has been explosive in recent decades to where now there are, in fact, about 706 medical schools in the Americas: 181 in North America, 190 in Central America and the Caribbean region and 335 in South America. This represents approximately 31% of the world total. Of these, 559 (79%) of the hemisphere´s medical schools are affiliated with FEPAFEM/PAFAMS.

Thus, our region is not immune to the proliferation of medical schools that has been present in recent decades worldwide coupled with a low efficacy in public health services. The increase in migration of medical personnel leads to a need to analyze and promote a "system of regional-international accreditation" (IAI), which likewise supports the development of quality assurance in medical education.

## Creation and aAchievements

Since its inception in 1962, and in accordance with its mission, FEPAFEM has focused on improving the quality of medical education. An initial step was to support national accreditation processes in member countries which were successfully developed locally and remain a key factor in improving the quality of institutions and its products^1^ ^,^ ^2^ . The application of evaluation mechanisms ^3^ ^,^ ^4^ and the discussed and accepted standards with necessary local adaptations has been a necessary step ^5^ , and all were done with the support of the World Federation for Medical Education (WFME) ^6^ . Their standards now are a common denominator for these ongoing processes. Essential contributions have been the proposals and work of the "Global Minimum Essential Requirements" (GMER) developed jointly with the Institute for International Medical Education (IIME)^7^.

## Geopolitics, migration and global practice:

The American continent, with an area of over 42 MM square kilometers is the second largest in the world, covering 8.3% of its total area, and accounting for about 13% of the world population. The region has extreme contrasts between sub regions and within the countries themselves, not only from the standpoint of socio-economic and political differences, but also from the perspective of health care and education-related systems.

A medical school is an institution that has the capacity to produce and disseminate knowledge, and the duty to reflect critically, focusing primarily on its responsibility to the society in which it acts. The same must be accompanied by autonomy and academic freedom, and its direction must ensure the quality of its graduates. In this regard, the issue of accreditation and public accountability arises as a systematic process of self-regulation for these institutions. Accreditation is essential, both for undergraduate and post-graduate students and for a lifetime of continuing education. In order to evaluate educational systems so that graduates achieve acceptable quality levels in different countries and regions, it is relevant to organize and structure a functional system that recognizes and harmonizes the necessary competencies to meet quality service needs in communities as well as apply principles of social responsibility, ethical and effective management, and emerging fundamentals for quality medical practice locally, regionally and globally.

Classically, the objectives of medical education have focused on basic fundamentals, namely establishing educational priorities and strategies for their achievement in medical schools, developing support resources, developing of admission policies consistent with the needs of the country or region, continuity in the phases of medical education, among others. However, the rapid changes brought about profound and important socio-political, economic and productive factors, as well as environmental considerations, directly affect the form and financing of medical practice, as well as the entire formative process of biomedical education. There are new needs, social and financial priorities that require the adoption of innovative policies and actions aimed at self-financing and development, especially those that harmonize medical education with health systems or with the absence of them.

Motivated by these needs, FEPAFEM's has among its objective to promote additions to a culture of quality that in medical school members translates into clear international recognition, based on what has been done nationally by our Associations in countries that use evaluation and accreditation as part of a change management strategy, along with modernization and improvements to the quality of medical education on the American continent to meet present and future challenges.

The concepts of quality medical education that have been intensely examined in recent times have changed from subjective appreciation to compliance with minimum requirements. It has also passed from a quantitative view to an added qualitative one that stresses competencies. The application of minimum requirements to evaluate the quality of medical education has had phases with various contents and strategies for the professor - services, biological, sociological and political, both preventive and curative, for both inpatient and outpatient settings, among others. It has also emphasized a focus on fundamental clinical areas, such as a focus on general and family care. An education based on profiles compatible with the roles and responsibilities, competencies and professional performance are required.

## An international accreditation initiative would have as basic objectives

To prepare doctors for the needs and expectations of the society in a globalized world.

To understand and harmonize the technological explosion with scientific and medical knowledge.

To instill abilities in physicians for lifelong learning, ethics and professionalism.

To ensure training in the new information technologies.

To build a system of medical education, and knowledge transfer I harmony with the changing conditions in the health care systems of our region.

To recognize that the capacity and will to change is within ourselves.

The challenge is putting it in practice.
